# Identification of Ceruloplasmin as a Gene that Affects Susceptibility to Glomerulonephritis Through Macrophage Function

**DOI:** 10.1534/genetics.116.197376

**Published:** 2017-04-24

**Authors:** Tai-Di Chen, Maxime Rotival, Ling-Yin Chiu, Marta Bagnati, Jeong-Hun Ko, Prashant K. Srivastava, Enrico Petretto, Charles D. Pusey, Ping-Chin Lai, Timothy J. Aitman, H. Terence Cook, Jacques Behmoaras

**Affiliations:** * Centre for Complement and Inflammation Research, Imperial College London, London W12 0NN, United Kingdom; † Department of Anatomic Pathology, Chang Gung Memorial Hospital, 33305 Taoyuan, Taiwan; ‡ Institut Pasteur, Unit of Human Evolutionary Genetics, Paris 75015, France; § Kidney Institute, Department of Nephrology, Chang Gung Memorial Hospital, 10591 Taipei, Taiwan; ** Division of Brain Sciences, Imperial College London, Hammersmith Hospital Campus, London W12 0NN, United Kingdom; †† Duke-NUS Medical School, National University of Singapore, Singapore 169857, Singapore; ‡‡ Renal and Vascular Inflammation Section, Imperial College London, London W12 0NN, United Kingdom; §§ Institute of Genetics and Molecular Medicine, University of Edinburgh, Edinburgh EH4 2XU, United Kingdom

**Keywords:** QTL, eQTL, fine mapping, glomerulonephritis, positional cloning, macrophages, Genetics of Immunity

## Abstract

Crescentic glomerulonephritis (Crgn) is a complex disorder where macrophage activity and infiltration are significant effector causes. In previous linkage studies using the uniquely susceptible Wistar Kyoto (WKY) rat strain, we have identified multiple crescentic glomerulonephritis QTL (*Crgn*) and positionally cloned genes underlying *Crgn1* and *Crgn2*, which accounted for 40% of total variance in glomerular inflammation. Here, we have generated a backcross (BC) population (*n* = 166) where *Crgn1* and *Crgn2* were genetically fixed and found significant linkage to glomerular crescents on chromosome 2 (*Crgn8*, LOD = 3.8). Fine mapping analysis by integration with genome-wide expression QTLs (eQTLs) from the same BC population identified ceruloplasmin (*Cp*) as a positional eQTL in macrophages but not in serum. Liquid chromatography-tandem mass spectrometry confirmed Cp as a protein QTL in rat macrophages. WKY macrophages overexpress Cp and its downregulation by RNA interference decreases markers of glomerular proinflammatory macrophage activation. Similarly, short incubation with Cp results in a strain-dependent macrophage polarization in the rat. These results suggest that genetically determined Cp levels can alter susceptibility to Crgn through macrophage function and propose a new role for Cp in early macrophage activation.

GLOMERULONEPHRITIS is often caused by immunecomplex-mediated inflammatory damage to the kidney. Recent genome-wide association studies (GWAS) revealed a strong genetic component with multiple loci, but the cellular mechanisms through which they modulate disease severity remain unknown (Gharavi *et al.* 2011; Lyons *et al.* 2012; Chung *et al.* 2014). The model of crescentic glomerulonephritis (Crgn) in the Wistar Kyoto (WKY) rat is a highly reproducible model that histologically resembles human focal and segmental necrotizing glomerulonephritis with crescent formation, as seen in antineutrophil cytoplasmic autoantibodies (ANCA)-associated vasculitis, lupus nephritis, and anti-glomerular basement membrane (anti-GBM) disease (Cook *et al.* 1999; Tam *et al.* 1999; Aitman *et al.* 2006; Behmoaras *et al.* 2008; Little *et al.* 2009; Smith *et al.* 2010; Reynolds *et al.* 2012). The WKY rat is uniquely susceptible to macrophage-dependent Crgn with crescent formation, macrophage infiltration, and proteinuria, only 10 days following the injection of nephrotoxic serum (NTS), a rabbit anti-rat GBM serum. Although this strain develops severe nephrotoxic nephritis (NTN) and progresses toward renal failure, another inbred strain, the Lewis (LEW) rat, which shares the same MHC haplotype, is resistant to NTN. We therefore took advantage of the [WKY × LEW] parental and segregating crosses to study the genetic components of Crgn in an MHC-independent way and identified susceptibility genes and cellular mechanisms underlying glomerular inflammation in Crgn (Aitman *et al.* 2006; Behmoaras *et al.* 2008, 2010; Deplano *et al.* 2013).

Macrophages are effector cells in human Crgn (Neale *et al.* 1988; Nikolic-Paterson and Atkins 2001; Kluth *et al.* 2004; Rees 2010), and our studies aiming to dissect the polygenic complex architecture of Crgn in the WKY rat led to the identification of genes that cause Crgn through regulation of macrophage activation and infiltration (Aitman *et al.* 2006; Behmoaras *et al.* 2008, 2010). The first genome-wide linkage analysis identified seven Crgn quantitative trait loci (Aitman *et al.* 2006) (QTL, *Crgn1-7*) with *Crgn1* on chromosome 13 and *Crgn2* on chromosome 16, both with LOD > 8, indicating very significant association with Crgn phenotypes. We have generated reciprocal congenic strains where *Crgn1* and *Crgn2* were introgressed into the genetic background of each strain (Behmoaras *et al.* 2010; D’Souza *et al.* 2013). Bone marrow transplantation experiments have confirmed that *Crgn1–7* contribute to glomerular crescent formation through macrophage activation (Behmoaras *et al.* 2010).

Furthermore, positional cloning studies led to the identification of variants in *Fcgr3* (Aitman *et al.* 2006) (*Crgn1*) and *JunD* (Behmoaras *et al.* 2008) (*Crgn2*) loci explaining 40% of the susceptibility to Crgn (Behmoaras *et al.* 2010) through macrophage function (Page *et al.* 2012; Deplano *et al.* 2013; Hull *et al.* 2013). Complementary to linkage studies, expression QTL (eQTL) approaches using macrophages from a segregating population from WKY and LEW rats identified genes that could also be targeted and reduce the severity of NTN in the WKY rat (Kang *et al.* 2014). Despite all these positional cloning and QTL studies, the remaining NTN susceptibility loci account for 60% of glomerular crescent formation, and the biological mechanisms through which they regulate Crgn remain to be elucidated.

In this study, we undertook a genetic approach aiming to fix the most significant Crgn QTL (*Crgn1* and *Crgn2*; LOD > 8) such that recombination will occur outside these genomic loci. We performed a restrictive genome-wide linkage analysis in a backcross (BC) population using single nucleotide polymorphisms (SNPs) derived from a custom-designed rat-specific RATDIV array (Rat Genome *et al.* 2013) and identified significant linkage to glomerular crescents on chromosome 2 (*Crgn8*, LOD = 3.8). We then applied a fine mapping strategy using integrative approaches combining genome-wide eQTLs in macrophages from the same population with quantitative trait transcript (QTT) analysis (Passador-Gurgel *et al.* 2007) focusing on the 1-LOD drop interval candidates. This prioritized ceruloplasmin (Cp), as the most significantly Crgn-associated transcript in macrophages, which is also expression and protein QTL. NTN-susceptible WKY rat macrophages overexpress Cp messenger RNA (mRNA) and protein levels and its knockdown leads to decreased macrophage-derived proinflammatory markers in Crgn. In keeping with this, short-time incubation of macrophages with Cp results in a genotype-dependent macrophage activation. RNA interference (RNAi) and Cp-stimulation experiments identified *Tnfa*, *Il1b*, *Mmp9*, and *Mt1* as Cp targets in macrophages, suggesting that targeting macrophage *Cp* expression could be important in attenuating glomerular inflammation in Crgn.

These results suggest that genetically determined Cp levels are associated with glomerulonephritis through macrophage function in the rat. They also highlight the previously unappreciated importance of Cp-mediated pathways in early macrophage activation, which is characterized by modulation of a subset of transcriptional markers of cell polarization. The exact mechanisms through which Cp regulates transcriptional programming of macrophages will help understanding the plasticity of these cells in inflammatory diseases.

## Materials and Methods

### Animals

**Figure 1:**
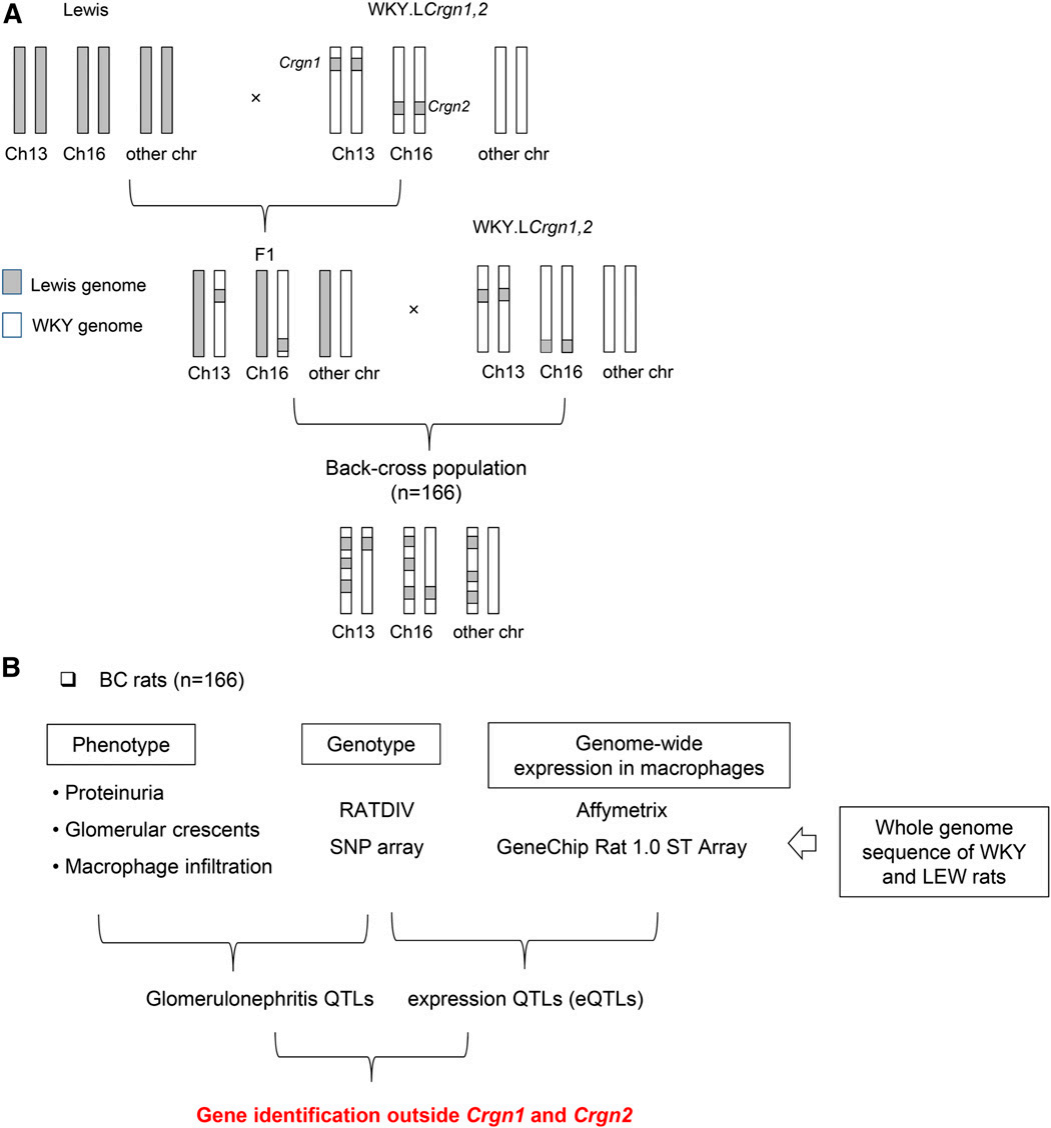
Fine mapping strategies in gene identification for crescentic glomerulonephritis. (A) Backcross (BC) breeding program using NTN-susceptible rats bicongenic for *Crgn1* and *Crgn2* (WKY.L*Crgn1,2*) and NTN-resistant Lewis (LEW) controls. (B) Fine mapping analysis by integrating Crgn QTL with genome-wide eQTL mapping from the BC population (*n* = 166). The whole genome sequencing of WKY and LEW rats (Illumina HiSeq 2000) is also used to eliminate false positives in the microarray analysis due to genetic variation that could affect probe intensity.

Wistar Kyoto (WKY/NCrl) and Lewis (LEW/Crl) rats were purchased from Charles River, United Kingdom. A total of 166 BC rats were produced by breeding LEW rats with the bicongenic WKY.L*Crgn1,2* as described in Figure 1. The F_1_ animals were backcrossed to the parental bicongenic WKY rats to obtain the BC rats. All procedures were performed in accordance with the United Kingdom Animals (Scientific Procedures) Act, 1986.

### NTN phenotypes in BC rats

NTN was induced in 12-week-old male BC rats by intravenous injection of 0.1 ml of NTS. Nine days later, urine was collected by placing the rats in metabolic cages for 24 hr with free access to food and water. Proteinuria was determined by the sulphosalicylic acid method. Ten days after NTN induction, rats were culled and kidneys were formalin fixed and paraffin embedded. To quantify the degree of histological injury in NTN, 4-μm formalin-fixed paraffin-embedded kidney sections were stained with H&E and periodic acid–Schiff. A total of 100 consecutive glomeruli were assessed in a blinded manner, and the number of severely crescentic glomeruli was reported as a percentage of total glomeruli examined. For macrophage infiltration, formalin-fixed paraffin-embedded kidney sections were stained with mouse monoclonal antibody to ED-1 (Serotec, Oxford, United Kingdom), followed by an HRP-labeled anti-mouse polymer development system (EnVision+ System-HRP, K4007, Dako, United Kingdom). The cellular infiltrate in 10 consecutive glomeruli was quantified using automated image analysis software (ImagePro Plus, Media Cybernetics, Bethesda, MD) and expressed as a percentage of total glomerular cross-sectional area.

### Genotyping and SNP filtering

The genotyping protocol is described in Kang *et al.* (2014). Briefly, total DNA was extracted from BC spleen samples by standard phenol–chloroform protocols. The custom-designed rat-specific RATDIV array (Rat Genome *et al.* 2013) was used for detection of ∼500,000 genome-wide single nucleotide polymorphisms (SNPs). For genotyping, genomic DNA was subjected to standard Affymetrix SNP6.0 GeneChip protocol according to the manufacturer’s instructions. A total of 250 ng DNA was used for *Nsp*I and *Sty*I digestions. Genotype calling was performed using apt-probeset-genotype from the Affymetrix Power Tools apt-1.14.3 with the optimized filter settings for the RATDIV array. For SNP filtering, we applied the following settings: FLD > 4, NO of AA calls >9/AB >3/BB >9, >99% call rate per SNP and hetSO ≥ 0 and revealed 274,339 high-quality SNPs.

For the vast majority of SNPs, the frequency of the LEW allele is ∼25%, consistent with the chosen BC strategy. SNPs where the frequency differs significantly from 25% reflect either congenic regions (*Crgn1* and *Crgn2*) or those that showed genotype calling failure, which were discarded from eQTL and QTL analysis. The imputation strategy is previously described in Kang *et al.* (2014). The WKY and LEW whole-genome sequence data (Illumina HiSequation 2000; >10× coverage for both strains) (Atanur *et al.* 2013) was then used to define the WKY and LEW alleles at each individual SNP, resulting in 278,124 SNPs that can discriminate both alleles. SNPs where the BRLMM clustering was not consistent with the breeding strategy (heterozygous frequency <0.35 or >0.65, presence of Lewis homozygous genotypes) were discarded, leaving 242,252 SNPs. Missing genotypes were then imputed using fastPhase with the two founder haplotypes as previously described (Kang *et al.* 2014). The total number of SNPs for QTL and eQTL analyses were 1974 following removal of those in complete linkage disequilibrium (*r*^2^ = 1).

### NTN QTL and eQTL mapping

NTN QTL mapping was performed for each phenotype separately by linkage analysis. LOD scores were obtained using R qtl package (scanone function) and significance threshold was assessed based on 1000 permutations. Boundaries of the *Crgn8* QTL were obtained by taking the 1-LOD drop interval around the linkage peak. For each gene in the 1-LOD drop interval, we then tested for correlations between gene expression and percentage of glomerular crescents, using linear models.

The eQTL mapping was performed as previously described (Kang *et al.* 2014) using ESS++ (Petretto *et al.* 2010; Bottolo *et al.* 2011). Fixed effects on each individual were added as covariates in the variable selection process to account for potential outliers or genotyping errors. A transcript was designated as an eQTL when it mapped with a probability of ≥0.8 to a genomic region of <10 Mb. When multiple SNPs are located within the 10-Mb windows, we refer to the SNP with the highest marginal probability in that region as the eQTL.

### Serum Cp quantity and activity measurements

Ceruloplasmin activity was measured in the serum of WKY and LEW rats before and after NTN induction using a colorimetric assay based on substrate oxidation (Sigma, St. Louis, MO, catalogue no. MAK177). Ceruloplasmin quantity was analyzed using the Rat Ceruloplasmin assay kit (AssayPro, St. Charles, MO, catalogue no. ERC4001-1) according to the manufacturer’s instructions.

### Cell culture, reagents, RNAi, and quantitative RT-PCR

Bone marrow-derived macrophages (BMDMs) were isolated and characterized as described previously (Lai *et al.* 2014; Behmoaras *et al.* 2015). Bone marrow cells were allowed to differentiate in Dulbecco’s modified Eagle’s medium (DMEM) (Thermo Fisher Scientific, Waltham, MA) containing 25 mM HEPES buffer (Sigma), 25% L929-conditioned medium, 25% fetal bovine serum (Labtech, batch 40811), penicillin (100 units/ml; Thermo Fisher Scientific) and streptomycin (100 μg/ml; Thermo Fisher Scientific), and cultured for 5 days in Petri dishes (Nunc). Purified ceruloplasmin was purchased from Enzo Life Sciences (Farmingdale, NY, catalogue no. ALX-200-089-M001).

For quantitative RT-PCR (qRT-PCR), total RNA was extracted from BMDMs using the TRIzol reagent (Invitrogen, Carlsbad, CA) according to the manufacturer’s instructions, and complementary DNA (cDNA) was synthesized using iScript cDNA Synthesis Kit (Bio-Rad, Hercules, CA). A total of 10 ng cDNA for each sample was used. All qRT-PCRs were performed on a ViaA 7 Real-Time PCR System (Life Technologies, Carlsbad, CA) using Brilliant II SYBR Green QPCR Master Mix (Agilent, Santa Clara, CA), followed by ViiA 7 RUO Software for the determination of Ct values. Results were analyzed using the comparative Ct method, and each sample was normalized to the reference mRNA of the *Hprt* gene, to account for any cDNA loading differences.

For RNAi, WKY BMDMs were replated in six-well plates (1 × 10^6^ cells per well) for an overnight period and transfected for 48 hr with On-Target Plus for human rat *Cp* (100 nM, Dharmacon SMART pool) or nontargeting small interfering RNA (siRNA) pool as the scrambled control siRNA using Dharmafect 1 (1:50, Dharmacon, Lafayette, CO) as a transfection reagent in Opti-MEM medium (Invitrogen). Primers and the siRNA sequence information are available upon request.

### Western blotting, ELISA, and Cp immunohistochemistry

For Western blot analysis, renal cortex tissue of day-10 nephritic kidneys from WKY and LEW rats were homogenized and lysed in RIPA buffer (Santa Cruz Biotechnology, Dallas, TX) with protease inhibitors, mixed with 2× Laemmli sample buffer (Bio-Rad), resolved by SDS/PAGE, transferred to polyvinylidene difluoride membranes, and subjected to immunoblotting with either rabbit monoclonal anti-ceruloplasmin (ab131220, Abcam, Cambridge, United Kingdom) or mouse monoclonal anti-β-actin (C4; Santa Cruz Biotechnology). Following incubation with secondary antibodies, the probed proteins were detected using SuperSignal West Femto Chemiluminescent Substrate (Thermo Fisher Scientific). For ELISA, TNFα (BD Biosciences, Billerica, MA) and IL-10 (Abcam) levels in BMDM culture supernatants were quantified using sandwich ELISA, according to the manufacturer’s instructions. Cp immunohistochemistry was performed on paraffin-embedded material sections with mouse anti-Cp (sc-135866, Santa Cruz Biotechnology) and developed using EnVision+ System-HRP (K4007, Dako). Pictures were taken by QImaging Retiga 2000R Scientific CCD camera using Image-Pro Plus version 7.0 software.

### Microarray expression profiling and quantitative proteomics

Sample preparation, microarray profiling, and data analysis for eQTL mapping in BC BMDMs were previously described in detail (Kang *et al.* 2014). For the parental strain BMDM microarrays, total RNA was extracted from WKY and LEW BMDMs (four biological replicates per strain; basal, 2-, 4-, and 8-hr LPS stimulation at 100 ng/ml) using the TRIzol method and purified using RNeasy Plus spin columns (Qiagen). A total of 100 ng of RNA was amplified, labeled, and hybridized to Rat Gene 1.0 ST arrays (Affymetrix, Santa Clara, CA) using the Ambion WT Expression Kit (Life Technologies) as per manufacturer instructions. CEL intensity files were produced using GeneChip Operating Software version 1.4 (Affymetrix, Santa Clara, CA) and quality tested using the Affymetrix Expression Console v1.1.2. Probe-level data were normalized using robust multichip average (RMA) (Bolstad *et al.* 2003; Irizarry *et al.* 2003). A custom definition file was created using up-to-date probe information (Dai *et al.* 2005) and filtered to exclude probes containing the 2,520,602 single nucleotide polymorphisms present between the WKY and LEW genomes. The moderated *t*-test with 40,000 permutations implemented in Statistical Analysis of Microarrays (SAM) version 3.0 was used to identify differentially expressed genes at a false discovery rate (FDR) threshold of 5% and timecourse analysis was performed using EDGE with 40,000 permutations and a 5% FDR threshold.

Detailed quantitative proteomics protocols by mass spectrometry in WKY and LEW BMDMs were previously described elsewhere (Rotival *et al.* 2015). Briefly, whole peptide dried extracts were resuspended in 14 μl of reconstitution buffer (0.1% trifluoroacetic acid containing 20 nm of an enolase digest). A total of 5 μl (peptides) was loaded in a liquid chromatography-tandem mass spectrometry (LC-MS/MS) system (nanoLC, Ultimate 3000 and LTQ-Orbitrap Velos mass spectrometer, Thermo Scientific). Raw data files were uploaded onto Progenesis QI for Proteomics software (Nonlinear Dynamics, 2014, version: 2.0.5387.52102). Chromatographic alignment (with additional manual manipulation), data normalization, and peak picking were performed by Progenesis QI. Mascot server (version 2.5.0) was used for peptide/protein identification as searched against the Uniprot Swissprot rattus norvegicus FASTA (downloaded June 6, 2014) which contained 7914 sequences.

### Data availability

WKY and LEW BMDM [basal and lipopolysaccharide (LPS)] microarray data set was deposited to ArrayExpress (accession no. E-MEXP-3469, https://www.ebi.ac.uk/arrayexpress/experiments/E-MEXP-3469/). Microarrays used for eQTL analysis including genotypes and phenotypes were also deposited to ArrayExpress (accession no. E-MTAB-2719, http://www.ebi.ac.uk/arrayexpress/experiments/E-MTAB-2719/). For the proteomics data set, results from Mascot searches were deposited into the ProteomeXchange Consortium (http://proteomecentral.proteomexchange.org) via the PRIDE partner repository with the data set identifier project accession PXD001269 and project DOI: 10.6019/PXD001269.

### Statistical analysis

Data are represented as mean ± 1 SD. All statistical analyses involving comparison between more than two groups were performed by ANOVA followed by Tukey’s multiple comparison post-test.

## Results

### Genome-wide linkage analysis in bicongenic rats

In previous studies, we have identified genes underlying *Crgn1* and *Crgn2* in the WKY NTN model. To allow locus-restrictive recombination, we took advantage of the previously generated bicongenic rats on a WKY background with introgression of LEW *Crgn1* and *Crgn2* (Behmoaras *et al.* 2010). The F_1_ rats between LEW and WKY.L*Crgn1,2* rats were backcrossed to the parental bicongenic to allow recombination to occur outside *Crgn1* and *Crgn2* loci (Figure 1A). NTN phenotypes such as percentage of glomerular crescents and macrophage infiltration as well as proteinuria were used in genome-wide linkage analysis (Figure 1B). As macrophages are the major drivers in crescentic glomerulonephritis (Nikolic-Paterson and Atkins 2001; Isome *et al.* 2004; Wang and Harris 2011), macrophage eQTLs from the same BC population were mapped and used for fine mapping together with whole-genome sequencing data (Figure 1B) available for parental WKY and LEW strains (Atanur *et al.* 2013).

### Identification of ceruloplasmin as a positional candidate for macrophage function in Crgn

**Figure 2:**
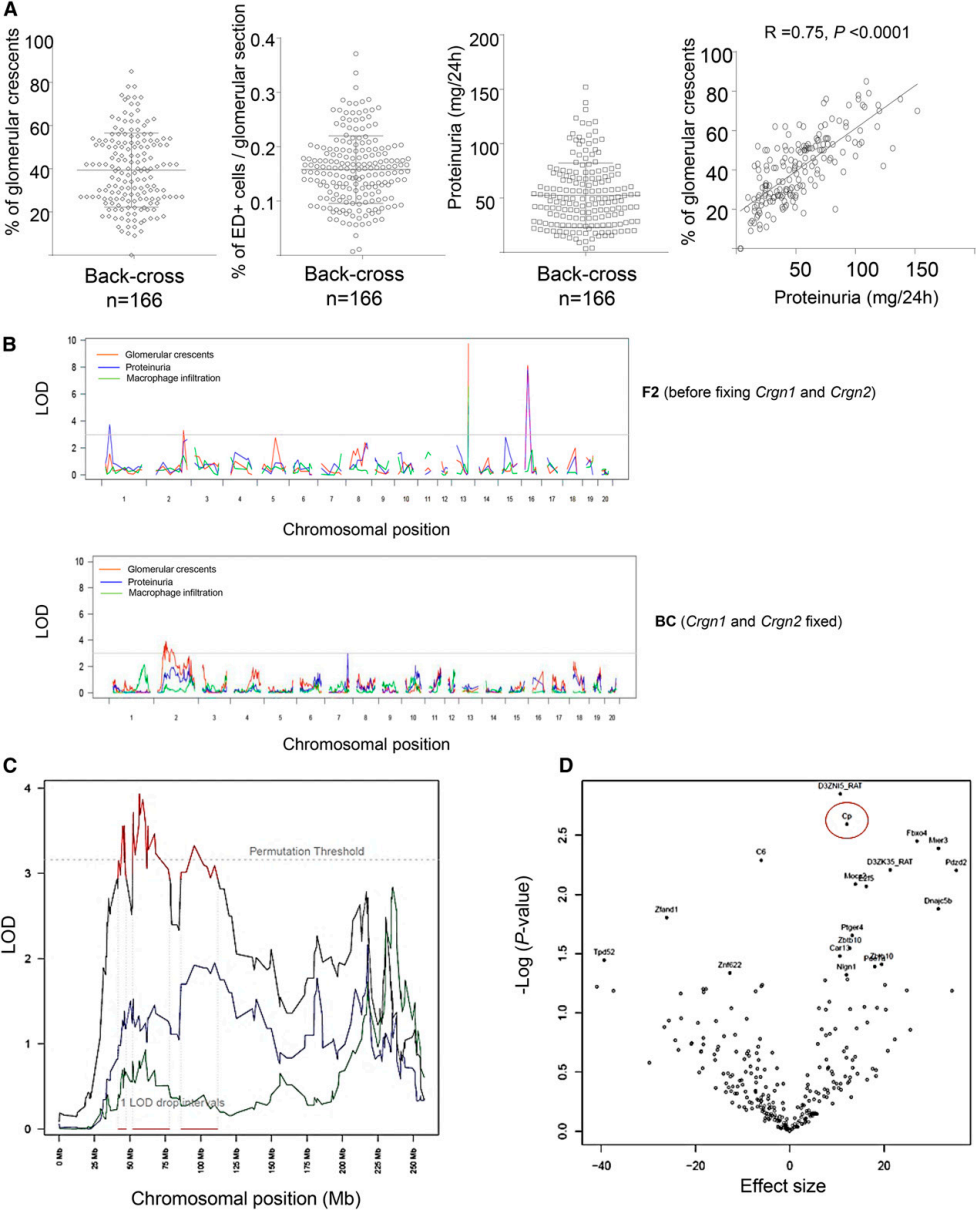
Susceptibility to nephrotoxic nephritis (NTN) when *Crgn1* and *Crgn2* are genetically fixed. (A) Percentage of glomerular crescents, ED-1^+^ macrophages per glomerular cross-section and proteinuria (milligrams/24 hr) in the BC population. Pearson correlation between percentage of glomerular crescents and proteinuria (milligrams/24 hr). (B) Genome-wide linkage of glomerulonephritis-related phenotypes in F_2_ (top panel) and BC (bottom panel). LOD scores for each phenotypes are represented (red, percentage of glomerular crescents; blue, proteinuria; and green, macrophage infiltration). (C) Chromosomal view of *Crgn8* on chromosome 2. Percentage of glomerular crescents, proteinuria, and macrophage infiltration are shown in black, blue, and green, respectively. The 1-LOD drop intervals are shown in red. (D) Volcano plot showing quantitative trait transcript (QTT) analysis within the 1-LOD drop interval (41.6–112 Mb). The *P*-value refers to the association with percentage of glomerular crescents, while the effect size denotes the effect of a twofold change in macrophage expression on the percentage of glomerular crescents. *Cp* (circled in red) is the strongest annotated QTT, which is also a *cis*-eQTL within the 1-LOD drop interval.

The BC population showed a wide range of quantitative phenotypes with a strong correlation between glomerular crescents and proteinuria (Figure 2A), suggesting that loci outside *Crgn1* and *Crgn2* contribute significantly to disease severity. As expected, genome-wide linkage analysis performed with SNPs on the original F_2_ population derived from WKY and LEW rats identified *Crgn1* and *Crgn2* with LOD > 6 (Figure 2B). When linkage analysis was performed in the BC population displaying genetically fixed *Crgn1* and *Crgn2*, this led to the identification of *Crgn8* on chromosome 2 (LOD = 3.8, *P* < 0.012 following 1000 permutations). Macrophage transcript expression of all genes located within the 1-LOD drop interval (41.6–112 Mb, Figure 2C) was tested for correlation with percentage of glomerular crescents by QTT analysis (Passador-Gurgel *et al.* 2007) (Figure 2D). Among all annotated genes within the 1-LOD drop, ceruloplasmin (*Cp*) expression in macrophages showed the most significant correlation with percentage of glomerular crescents (Figure 2D and Table 1). *Cp* is strongly *cis*-regulated (*R*^2^ = 0.4, *P* = 2.59 × 10^−23^) and is one of three *cis*-eQTL genes (together with *C6* and *Pde7a*) mapped with high confidence (marginal probability > 0.8) within the peak of linkage. Of these genes, macrophage *Cp* is the only transcript that also shows a significant correlation with percentage of glomerular crescents (Table 1). Taken together, these results suggested that macrophage *Cp* expression could partly explain susceptibility to Crgn. We thus hypothesized that genetically determined Cp levels could be essential for transcriptional activation in macrophages.

**Table 1:** eQTLs within the 1-LOD drop interval on *Crgn8*

Gene	Best SNP position (bp)	MP	Gene start (bp)	Gene end (bp)	*R*^2^	*P*	*R* (crescents)	*P*_adj_ (crescents)
*C6*	*52574178*	0.95	53691288	53764541	0.53	9.85 × 10^−33^	−0.20	0.08
*D3ZNI5_RAT*	*89758582*	0.49	95174024	95176430	0.43	2.18 × 10^−25^	0.23	0.03
***Cp***	***95386102***	**0.91**	**105086278**	**105145860**	**0.40**	**2.59 × 10^−23^**	**0.21**	**0.04**
*Fabp4*	*95386102*	0.55	93536110	93540771	0.24	1.37 × 10^−12^	−0.13	0.47
*Car13*	*106915624*	0.73	88271908	88309336	0.27	6.07 × 10^−15^	0.15	0.40
*Ptger4*	*61068139*	0.51	54423275	54434520	0.21	1.41 × 10^−11^	0.16	0.29
*Znf622*	*61068139*	0.35	77369798	77585703	0.10	5.26 × 10^−06^	−0.14	0.46
*RGD1310081*	*57201899*	0.67	57835595	57879494	0.10	3.34 × 10^−06^	0.14	0.47
*Car2*	*82416512*	0.18	88077084	88092256	0.10	3.86 × 10^−06^	−0.09	0.85
*D3ZK35_RAT*	*56704838*	0.56	57781798	57811897	0.21	1.47 × 10^−11^	0.19	0.09
*Zfp458*	*109800573*	0.34	86170500	86179868	0.12	1.07 × 10^−06^	−0.06	0.85
*Pde7a*	*106915624*	0.95	104339910	104429820	0.32	5.78 × 10^−18^	0.15	0.45
*Snx16*	*104467862*	0.28	93274839	93296187	0.19	2.79 × 10^−11^	−0.13	0.47
*Zfand1*	*95386102*	0.51	93397683	93406694	0.25	1.35 × 10^−13^	−0.17	0.22
*Mier3*	*46893067*	0.34	42999528	43024481	0.14	1.29 × 10^−08^	0.20	0.07
*Myo10*	*67448697*	0.26	77183028	77385896	0.12	5.35 × 10^−07^	0.09	0.85
*Golph3*	*52381864*	0.33	61789212	61817177	0.10	4.00 × 10^−06^	0.03	0.85

For each gene, only the transcript with the most significant eQTL is reported. SNPs with a marginal probability (MP) of association of at least 80% were considered as eQTLs. *Cis*-eQTLs are defined as the SNP with highest marginal probability located within 10 Mb of the gene. *P*-value of the eQTL is calculated with a univariate *t*-test. *R* and the adjusted *P*-value (*P*_adj_) refer to the correlation of the transcript with glomerular crescents. *Cp* is shown in bold as the only eQTL with MP > 80% correlating significantly with the percentage of crescents.

### Ceruloplasmin is a macrophage expression and protein QTL

**Figure 3:**
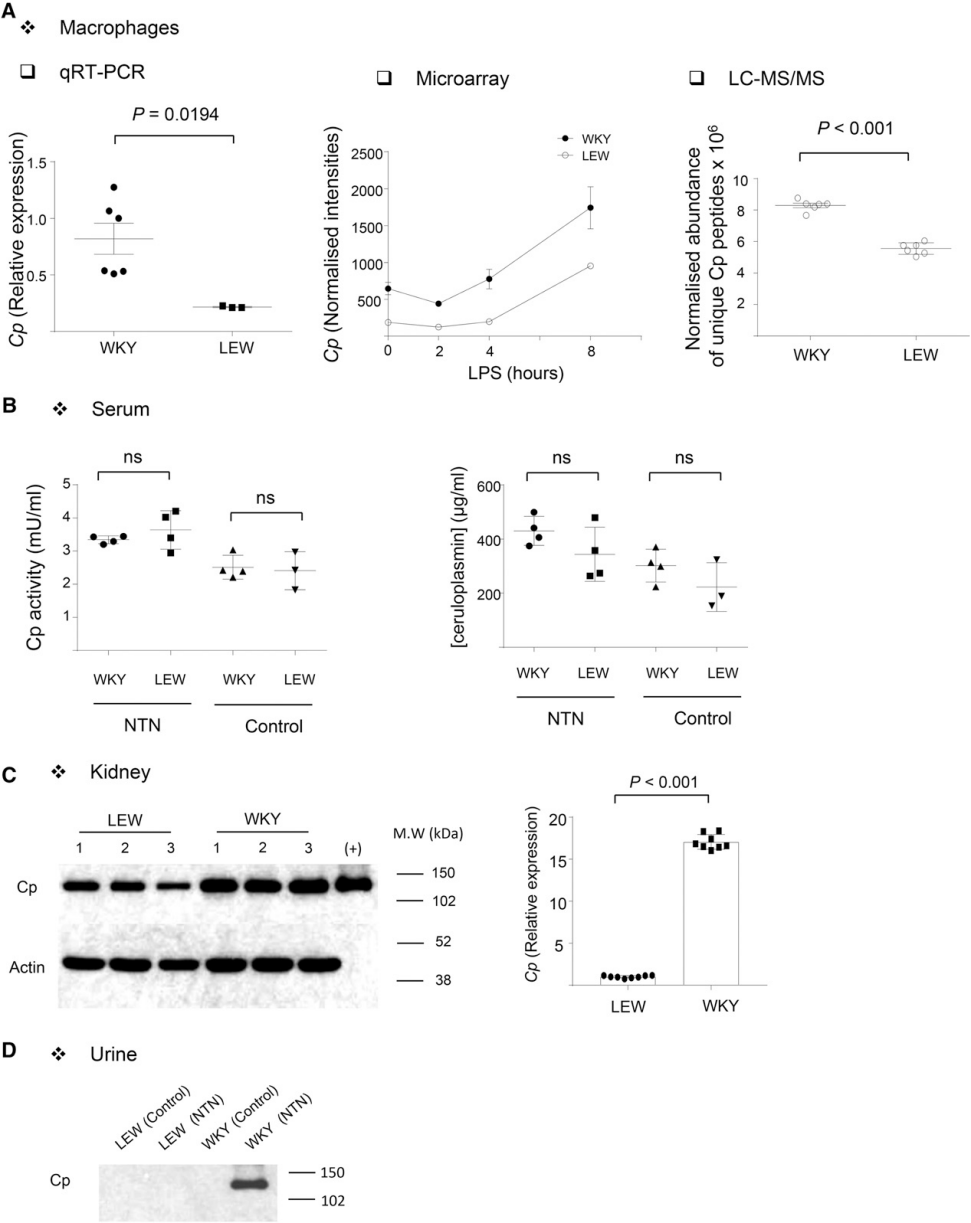
Ceruloplasmin is an eQTL in macrophages but not in serum. (A) qRT-PCR, microarray (*n* = 4 rats/strain; normalized intensities are shown in basal and 2, 4, and 8 hr 100 ng/ml LPS-stimulated), and quantitative proteomics (LC-MS/MS, *n* = 3 rats/strain) in WKY and LEW BMDMs show Cp overexpression in WKY rats. (B) Ceruloplasmin activity (left panel) and quantity (right panel) in WKY and LEW serum before and after NTN induction. At least *n* = 3 biological replicates were used in each group. (C) Cp Western blot (left panel) and qRT-PCR (right panel) in LEW and WKY NTN kidneys. The positive control for the Western blot is a proteinuric urine sample from WKY rats following NTN, and the numbers denote the biological replicates from each strain. For qRT-PCR, *n* = 4 rats/group were used. (D) Cp Western blot in normal and nephritic LEW and WKY urine.

To confirm that *Cp* is an eQTL, we cultured BMDMs from WKY and LEW rats and measured *Cp* mRNA by qRT-PCR (Figure 3A). This analysis confirmed *Cp* as a *cis*-eQTL. This was further consolidated in a microarray analysis using WKY and LEW BMDMs in basal and LPS-stimulated (2, 4, and 8 hr) states (Figure 3A). Quantitative proteomics by LC-MS/MS in WKY and LEW BMDMs showed that the difference in mRNA levels is further associated with a significant difference in protein levels (Figure 3A), suggesting that Cp is a protein QTL in primary rat macrophages. Because ceruloplasmin is an acute-phase plasma protein made principally by hepatocytes and activated monocytes/macrophages (Yang *et al.* 1986; Vassiliev *et al.* 2005), we investigated whether the genetic control of its protein levels and activity was conserved in the serum. Interestingly, neither Cp quantity nor activity in the serum was significantly different between WKY and LEW strains (Figure 3B), suggesting that the genetic control of macrophage Cp levels could partly explain the pathophysiology of NTN in the rat. Supporting the latter, Cp-positive macrophages were detected by immunohistochemistry in the glomeruli and interstitium of nephritic WKY kidneys (Supplemental Material, Figure S1). To further confirm the pathological relevance of Cp differential expression in Crgn, we compared Cp expression levels between WKY and LEW nephritic renal cortex and showed significantly higher Cp protein and mRNA levels in WKY rats (Figure 3C). Cp was also detected in the proteinuric urine of WKY rats following NTN induction (Figure 3D). Taken together, these results show that genetically determined Cp mRNA and protein levels in macrophages are also conserved in the kidneys following NTN induction, supporting further the association between Cp and Crgn.

### Ceruloplasmin is a determinant of macrophage activity in the rat

**Figure 4:**
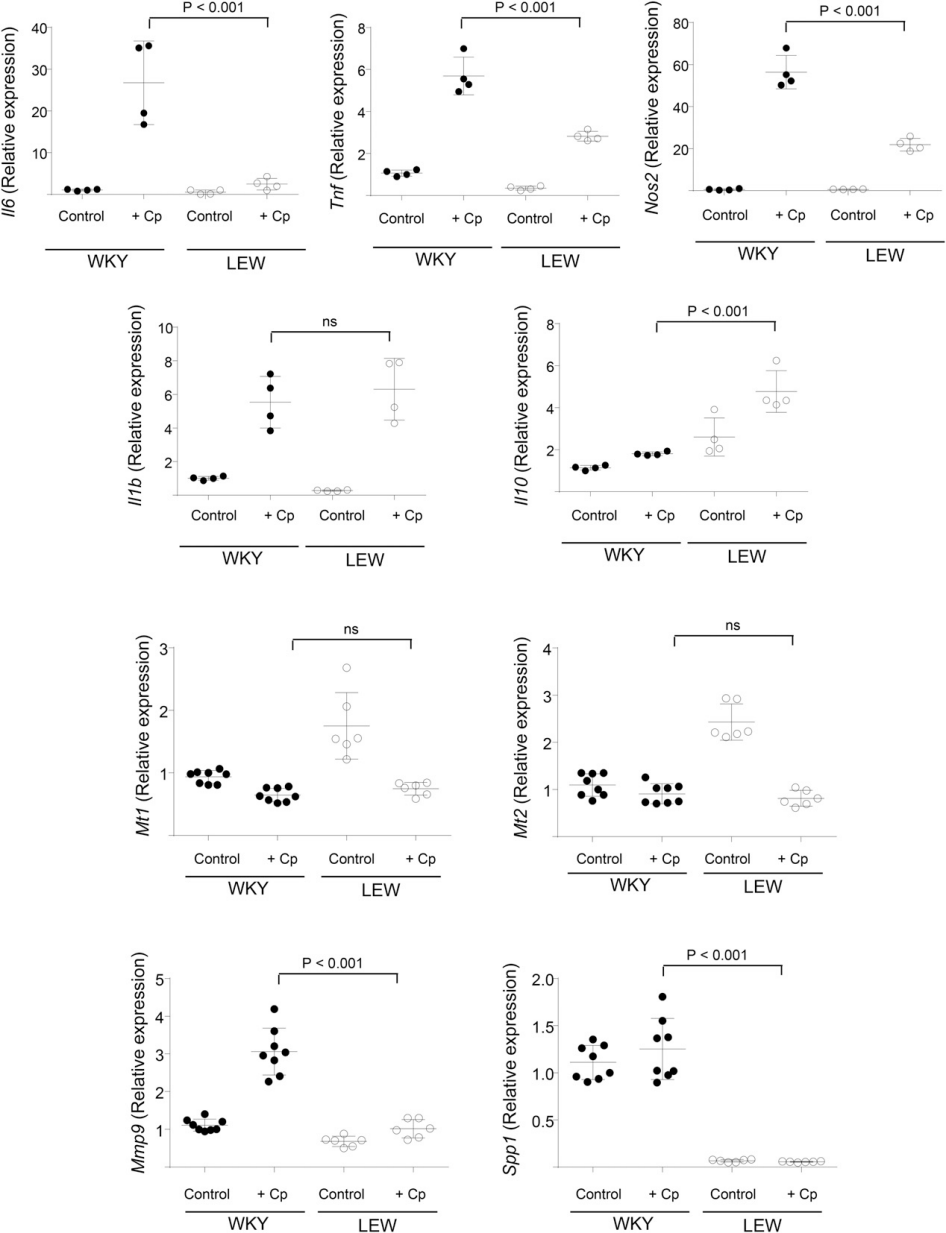
Cp addition polarizes macrophages. BMDMs from WKY and LEW rats were left unstimulated (control) or stimulated with Cp (15 µg/ml) for 3 hr. *Il6*, *Tnf*, *Nos2*, *Il1b*, *Il10*, *Mt1*, *Mt2*, *Mmp9*, and *Spp1* mRNA levels were measured by qRT-PCR. For simplicity, the statistic comparisons between [WKY + Cp] and [LEW + Cp] are shown on the graph. Cp stimulation resulted in a significant upregulation of *Il6*, *Tnf*, *Nos2*, *Il1b*, and *Mmp9* in WKY BMDMs (*P* < 0.001) and *Tnf*, *Nos2*, *Il1b*, and *Il10* in LEW BMDMs (*P* < 0.001). *Mt1* and *Mt2* were significantly downregulated in LEW BMDMs (*P* < 0.001). Results were analyzed by ANOVA, followed by Tukey’s multiple comparison test.

To gain insights into the role of Cp in macrophage function, we incubated WKY and LEW BMDMs with purified Cp for 3 hr. We reasoned that if Cp had a transcriptional effect on macrophage activation, this should result in early activation of transcripts well-described in macrophage activation. Notably, Cp addition resulted in a significant upregulation of *Il6*, *Nos2*, *Tnf*, *Il1b*, and *Il10* expression levels in macrophages from at least one strain of rat (Figure 4). There was a genotype-dependent expression of these markers as the magnitude of *Il6*, *Tnf*, and *Nos2* expression levels was significantly higher in NTN-susceptible WKY BMDMs when compared with LEW ones (Figure 4). In keeping with this, *Il10*, the anti-inflammatory macrophage marker, was expressed at relatively higher levels in LEW BMDMs in response to Cp stimulation. Notably, when we measured the secreted levels of TNFα and IL-10, we found a marked increase in TNFα secretion following incubation with Cp in WKY BMDMs only (Figure S2). Because ceruloplasmin is a major copper-carrying protein, we tested the expression of intracellular proteins such as metallothioneins (*Mt1* and *Mt2*), previously described in copper homeostasis (Suzuki *et al.* 2002) and in scavenging macrophage superoxide radicals (Irato *et al.* 2001). As opposed to the proinflammatory macrophage markers, *Mt1* and *Mt2* were down-regulated by Cp addition. WKY and LEW BMDMs showed a difference in basal levels of both metallothioneins (Figure 4). Glomerular crescent formation in Crgn is partly dependent on infiltrating macrophages expressing *Mmp9* and osteopontin (*Spp1*) (Triantafyllopoulou *et al.* 2010), which led us to measure the expression of these transcripts following addition of exogenous Cp. Purified Cp addition resulted in genotype-dependent increase in *Mmp9* mRNA levels while it had no effect on *Spp1*, despite a significant difference in basal BMDM *Spp1* mRNA levels between LEW and WKY rats (Figure 4).

**Figure 5:**
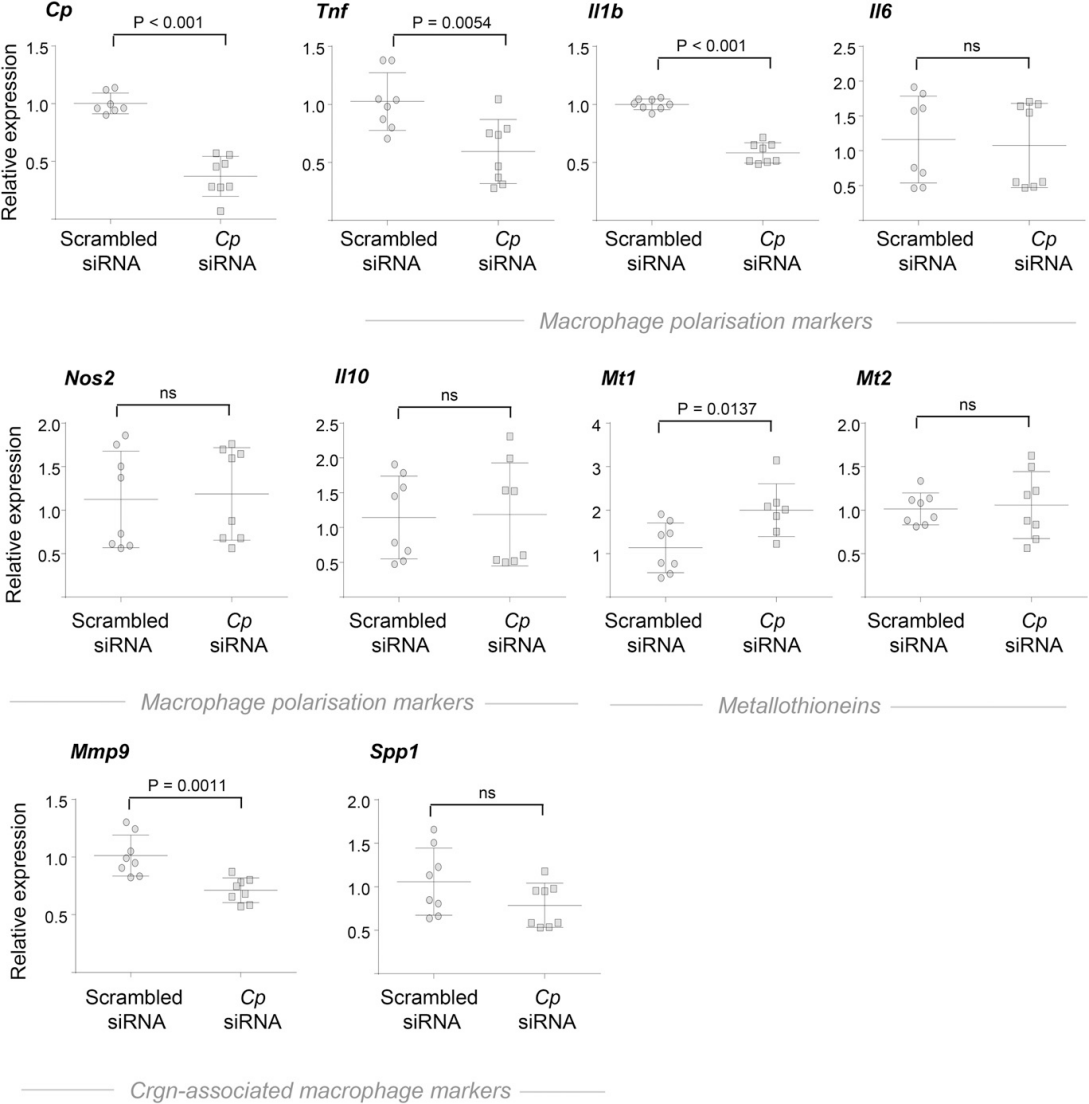
Cp knockdown and macrophage activation in rat BMDMs. WKY BMDMs were incubated with either scrambled or Cp siRNA for 48 hr and stimulated with LPS for 3 hr. *Cp*, *Tnf*, *Il1b*, *Il6*, *Nos2*, *Il10*, *Mt1*, *Mt2*, *Mmp9*, and *Spp1* mRNA levels were measured by qRT-PCR.

After establishing the polarizing effect of Cp addition, we investigated whether *Cp* knockdown recapitulated these results. *Cp* knockdown in WKY BMDMs, which overexpress the protein, led to a significant downregulation of *Tnfa*, *Il1b*, and *Mmp9*, whereas there were no significant changes in *Il6*, *Nos2*, and *Il10* levels (Figure 5). Furthermore, *Cp* knockdown was followed by a significant upregulation of *Mt1*, in line with the downregulation observed following incubation with Cp. In summary, *Tnfa*, *Il1b*, *Mmp9*, and *Mt1* were confirmed as Cp targets by using complementary approaches (Cp stimulation and RNAi) during early activation of primary rat macrophages.

## Discussion

During the development of crescentic glomerulonephritis, the major pathogenic event that causes crescent formation is the rupture of glomerular capillaries, which allows a relatively early macrophage infiltration into the Bowman’s space. There have been numerous reports showing that macrophage activity and numbers are critical in the inflammatory phase of Crgn (Duffield *et al.* 2005; Wang *et al.* 2007; Wang and Harris 2011) and our group has contributed to the identification of genetic and epigenetic determinants of macrophage function, which associate with susceptibility to Crgn in rats and humans (Aitman *et al.* 2006; Behmoaras *et al.* 2008, 2010; Page *et al.* 2012; Deplano *et al.* 2013; Hull *et al.* 2013; Kang *et al.* 2014; Rackham *et al.* 2017).

The identification of the most significant QTL and the underlying genes in the WKY NTN model, led us to develop single and double congenic animals where either *Crgn1* and/or *Crgn2* were introgressed into WKY or LEW genetic backgrounds (Behmoaras *et al.* 2010; D’Souza *et al.* 2013). These congenic strategies, combined with bone marrow transplantation studies, allowed us to conclude that after exclusion of *Crgn1* and *Crgn2*, NTN susceptibility accounts for ∼60% of the total variance in glomerular inflammation and is dependent on macrophage function (Behmoaras *et al.* 2010). In the current study, we have performed genome-wide linkage analysis using a BC population where segregation occurred independently from *Crgn1* and *Crgn2*. This approach identified a significant QTL on chromosome 2 (*Crgn8*), which was not present in the original F_2_ population used (Aitman *et al.* 2006 and Figure 2B), where the remaining loci were designated as *Crgn3–7*. One explanation for this is a possible interaction between *Crgn1* and/or *Crgn2* with *Crgn3–7*. A recent comprehensive QTL study in yeast showed that for the majority of the traits studied, one or a few additive QTL of large effects were observed together with many other QTL and QTL–QTL interactions of small effects (Bloom *et al.* 2015). It is therefore likely that *Crgn8* is a new NTN QTL, which shows no interactions with *Crgn1* and/or *Crgn2* as opposed to *Crgn3–7*.

The usage of the rat as a model organism for renal translational research has been recently proven insightful (Mamenko *et al.* 2016). Our fine mapping strategy based on integrative systems biology approaches (Moreno-Moral *et al.* 2017) prioritized ceruloplasmin as a positional candidate that regulates macrophage function in Crgn. *Cp* mRNA levels in macrophages correlate with the percentage of glomerular crescents and in addition to transcript levels, Cp protein levels are also genetically determined in the rat. Ceruloplasmin is the main mammalian copper transporter and an acute-phase plasma protein produced by hepatocytes and by interferon-γ-stimulated monocytes (Yang *et al.* 1986; Mazumder *et al.* 1997; Vassiliev *et al.* 2005). Cp plasma level nearly doubles in response to inflammation or infection (Gitlin 1988). Ceruloplasmin exerts a ferroxidase activity that converts Fe^2+^ to Fe^3+^, at the expense of O_2_, playing an important role in iron metabolism and transport through accelerating binding of iron by apotransferrin (Osaki *et al.* 1966). In addition, through its ferroxidase activity, ceruloplasmin inhibits ferrous ion-mediated production of reactive oxygen species, indicative of an antioxidant activity. In keeping with its antioxidant properties, macrophage-derived ceruloplasmin contributes importantly to protection against inflammation and tissue injury in acute and chronic experimental colitis (Bakhautdin *et al.* 2013). When exogenous Cp is added to WKY and LEW rat BMDMs, this stimulates WKY macrophages to produce significantly higher levels of proinflammatory cytokines such as TNFα, which is an important proinflammatory driver in the rat NTN model (Khan *et al.* 2005). WKY BMDMs have been previously described as having an M1-like phenotype such as enhanced superoxide production (D’Souza *et al.* 2013) and HIF-1-mediated glycolytic transcriptome (Rotival *et al.* 2015) when compared with macrophages from the NTN-resistant LEW rats. While Cp-mediated M1-like macrophage activation argues against its protective antioxidant properties, the role of Cp in iron efflux could provide a possible mechanism for the Cp-dependent proinflammatory macrophage function in the WKY rat. Cp stimulates iron release from macrophages under hypoxic conditions, possibly by generating a negative iron gradient (Sarkar *et al.* 2003). Interestingly, our LC-MS/MS results have shown that a great majority of the proteins belonging to iron metabolism are differentially produced between WKY and LEW BMDMs, suggestive of a general dysregulation of the intracellular iron metabolism in WKY macrophages. The strong metabolic shift toward HIF-1-mediated glycolysis specifically occurring at transcriptomic level during the differentiation of WKY BMDMs (Rotival *et al.* 2015) also argues in favor of a perturbed iron homeostasis in this strain, given the strong link between iron homeostasis and hypoxia-inducible transcription factors (Peyssonnaux *et al.* 2007). Hence the results showing the Cp-dependent increased proinflammatory macrophage activation in WKY BMDMs could be partly attributed to the role of Cp in regulating iron homeostasis. The role of iron in proinflammatory macrophage function was indeed established *in vivo* (Sindrilaru *et al.* 2011) and macrophage polarization is thought to be under the regulation of iron trafficking and metabolism in macrophages (Cairo *et al.* 2011). In addition to the iron link, a possible proinflammatory role of Cp through nitric oxide synthase activity and cytokine secretion was suggested in microglial cells of the brain (Lee *et al.* 2007; Lazzaro *et al.* 2014).

Ceruloplasmin has been previously suggested as a physiologic inhibitor of myeloperoxidase (MPO) (Segelmark *et al.* 1997). WKY rats immunized with MPO develop experimental autoimmune vasculitis (EAV) (Little *et al.* 2009), which could suggest a role of Cp in the pathophysiology of EAV. However, the mechanisms by which an increased Cp level in macrophages could lead to anti-MPO-mediated renal damage remain to be elucidated. Interestingly, when used in combination with other markers, urinary Cp levels were found to be a predictor of the activity of lupus nephritis in patients (Brunner *et al.* 2012). In summary, genetically determined macrophage Cp levels could define a macrophage activity that explains susceptibility to Crgn, and further studies aiming to understand the role of Cp in human macrophages will shed light into mechanisms of macrophage-dependent crescentic glomerulonephritis.

## Supplementary Material

Supplemental material is available online at www.genetics.org/lookup/suppl/doi:10.1534/genetics.116.197376/-/DC1.
