# Supplementary material for: Identification of Ceruloplasmin as a Gene that Affects Susceptibility to Glomerulonephritis Through Macrophage Function
Source: Genetics. 2017 Apr 24;206(2):1139–51. doi: 10.1534/genetics.116.197376 (PMC5499168; doi:10.1534/genetics.116.197376)
Supplement: Supplementary file 1 [file 1139FigureS1.pptx]

## Slide 1
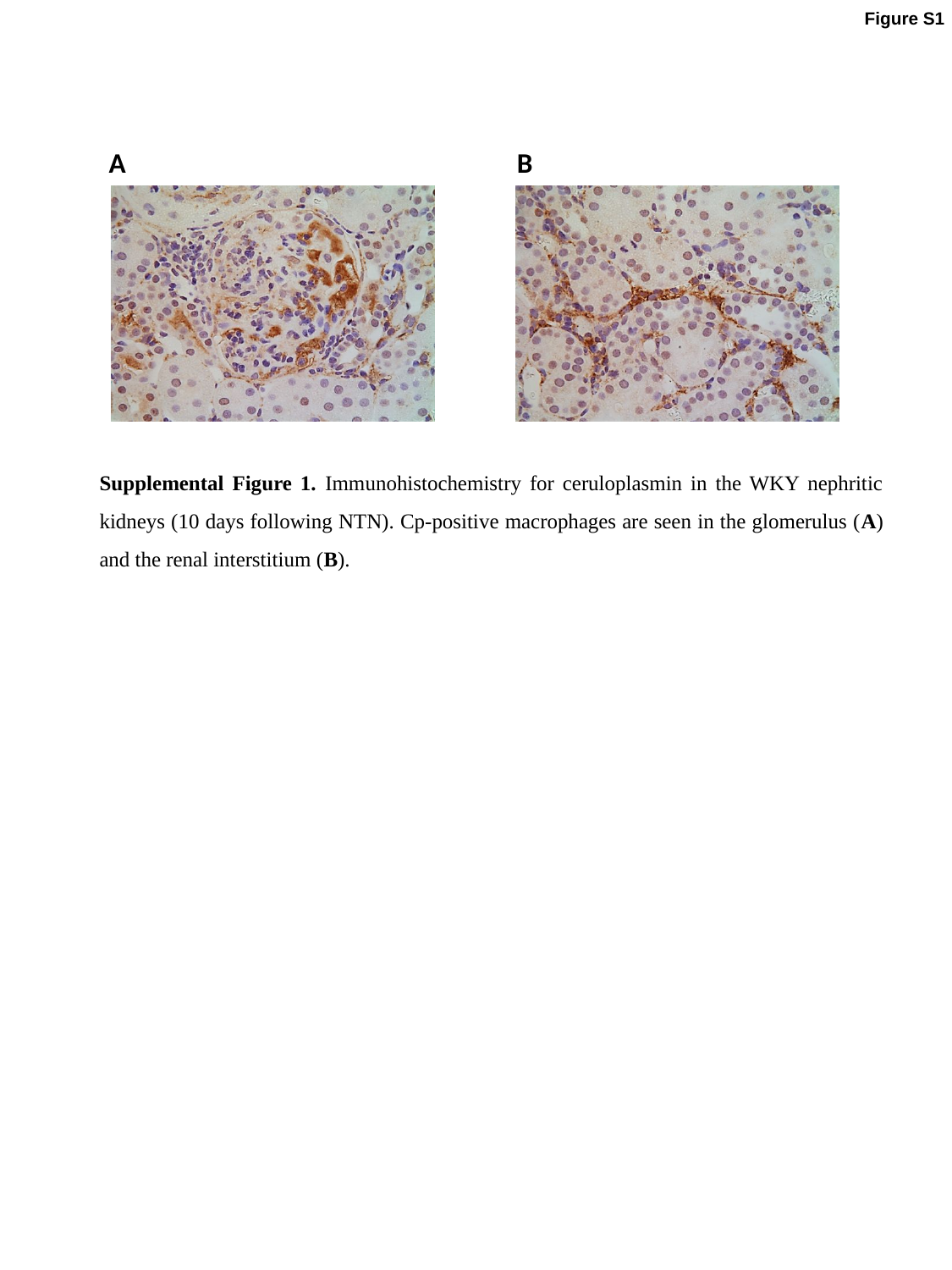

Figure S1
A
B
Supplemental Figure 1. Immunohistochemistry for ceruloplasmin in the WKY nephritic kidneys (10 days following NTN). Cp-positive macrophages are seen in the glomerulus (A) and the renal interstitium (B).
