# Supplementary material for: Identification of Ceruloplasmin as a Gene that Affects Susceptibility to Glomerulonephritis Through Macrophage Function
Source: Genetics. 2017 Apr 24;206(2):1139–51. doi: 10.1534/genetics.116.197376 (PMC5499168; doi:10.1534/genetics.116.197376)
Supplement: Supplementary file 2 [file 1139FigureS2.pptx]

## Slide 1
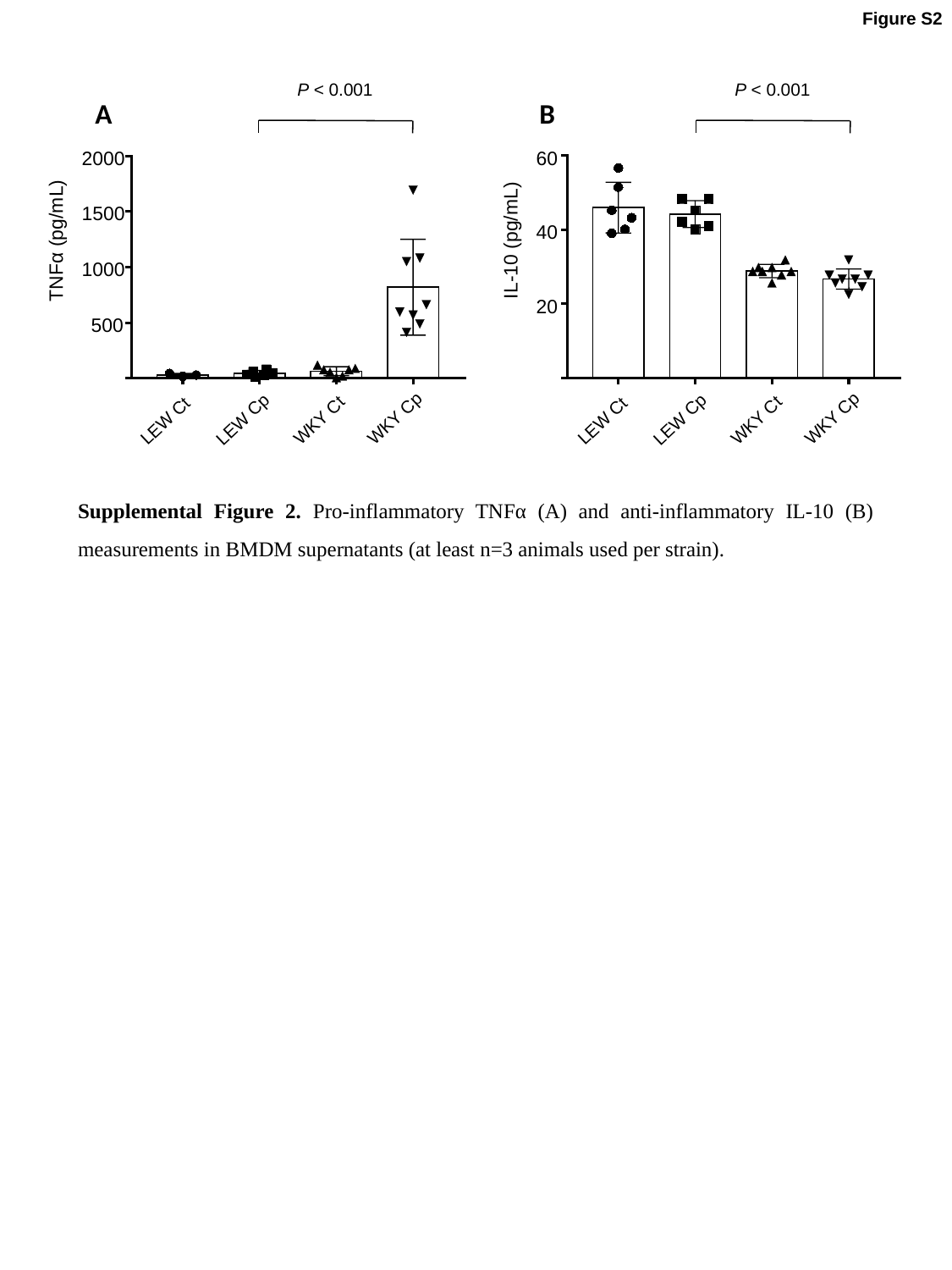

Figure S2
P < 0.001
P < 0.001
A
B
2000
60
1500
40
IL-10 (pg/mL)
TNFα (pg/mL)
1000
20
500
LEW Ct
WKY Cp
LEW Ct
WKY Cp
WKY Ct
WKY Ct
LEW Cp
LEW Cp
Supplemental Figure 2. Pro-inflammatory TNFα (A) and anti-inflammatory IL-10 (B) measurements in BMDM supernatants (at least n=3 animals used per strain).
